# Dental Materia Medica

**Published:** 1866-07

**Authors:** Geo. Watt


DENTAL REGISTER.

VOL. XX.] JULY 1866. [NO. 7.

### Original Communications.

### DENTAL MATERIA MEDICA.

BY GEO. WATT.

### ACONITE.

THIS is the *Aconitum Napellus* of Linnæus, and is ordinarily called "Monkshood" or "Walfsbane." Its botanic characteristics, and the various chemical compounds which it contains, are interesting; but a detail of them would occupy too much space for our present purpose. Its physiological effects, its uses, and the officinal preparations containing its active principles, and which may be useful to the dentist, will prove sufficient here. The dentist has never a call for its internal use; hence its topical action will be mainly, if not wholly considered.

PHYSIOLOGICAL EFFECTS.—These are striking and peculiar. Small quantities of the root, leaves, tincture, or extract, taken into the mouth, cause a tingling and benumbed sensation in the tongue and lips, which lasts for hours. By the use of larger quantities, the sensation is extended to the throat; and from its internal use, the same feelings extend to the extremities and throughout the system. Applied to or near the eye, it contracts the pupil. If used locally for facial neuralgia, it is prudent to mention this to the patient.

USES.—As it benumbs, or lessens the normal sensibility of nerves, it would seem to be adapted to the relief of exalted sensibility, or neuralgic pain. Experience teaches us that it is, in this respect, invaluable. Pereira says, "In *neuralgia*, no remedy will, I believe, be found equal to it. One application of the tincture produces some amelioration, and, after a few times' use, it frequently happens that the patient is cured. In some cases the benefit seems almost magical." But neuralgia, as a technical term, does not define a distinct and specific pathological condition; hence, we must not be surprised if the aconite sometimes fails to give such prompt relief in cases which we regard as neuralgic. But as its local use is not at all likely to do harm, it may be resorted to, even in doubtful cases; and, when it proves beneficial, the relief is usually so prompt as to gratify the applier, and delight the patient. When the pain results from acute inflammation, it is not very likely to give permanent relief; yet, in the treatment of dental periostitis, it is often valuable as a controller of pain, while the inflammation is conducted to one of its ordinary terminations. It is often more efficient, when applied over a nerve-trunk, than immediately over the seat of pain at the sentient extremities of the nerve. I have never tried it as an application to exposed and painful dental pulps; but where the exposure is so complete as to allow intimate contact, it would probably afford speedy relief. It often promptly allays the morbid sensibility of dentine, especially when the sensitiveness is superficial.

ADMINISTRATION.—It is best for dentists to rely on but two preparatiens of aconite, the *tincture*, and the *alcoholic extract*. The tincture is sufficiently active for topical use, as an embrocation. It may be applied as a liniment over the seat of pain, or over nerve-trunks leading to it. The alcoholic extract may be used as an ointment, by combining one part of it with two of lard or simple cerate. Or it may be spread on adhesive plaster, and applied where desired. Neither the tincture nor the extract should be used on abraded surfaces.

ACONTINE is an alkaloid substance contained in the plant, of which it appears to be the active principle. Its effects are like those of the root and leaves, but are very much more powerful. Indeed, its action is so energetic, that it is scarcely safe in any hands; and as the officinal preparations mentioned above are sufficiently active for all purposes, they should be relied on to the exclusion of acontine.

### BELLADONNA.

This plant is commonly called the *deadly nightshade.* The dentist never has occasion to prescribe it internally. Used locally, it often relieves neuralgic pains very promptly; but, I think it is less reliable than aconite. The solid extract dissolved in water, or the fluid extract, will be found convenient in dental practice. Either may be freely applied in facial neuralgia, over the cheek and jaws of the affected side, with decided relief, in many cases. The pupil of the eye will be dilated; which might frighten the patient, unless previously explained. I frequently prescribe, as a liniment for such cases, the following:

℞. Fluid Extract of Belladonna, Tincture
of Opium, each, - - - - 1 ounce.
Chloroform, - - - - - 3 drams.

Mix and use as a liniment on the affected side.

### VERATRIA.

This is a vegetable alkaloid, obtainable from most druggists, for which dentists may find occasional use. It may be applied externally to accomplish the same purpose for which the two preceeding medicines are recommended. Its local action is that of an irritant and benumber. Applied to the skin, as an ointment, it causes a tingling and burning sensation in the part, and sometimes in organs quite distant. It often relieves neuralgic pain with great promptness; but it is not so reliable as aconite. When the latter is not at hand or attainable, the veratria may prove highly useful. It may be made into an ointment, by combining twenty to forty grains of it with an ounce of lard or simple cerate.

### TOBACCO.

But little need be said to dentists in regard to the medical properties of this stinking weed. The entire plant is poisonous, and is of no use, in any way, to the dental surgeon. It is true that toothache is sometimes temporarily relieved by tobacco smoke, and may be suspended by the use of nicotin, or nicotianin; but as the latter two are not safe, and the former not decent, there is no call for their use, especially as more efficient remedies, compatible with civilization and good morals, are nearly always accessible.

